# Sex Differences in the Neural Correlates of Specific and General Autobiographical Memory

**DOI:** 10.3389/fnhum.2016.00285

**Published:** 2016-06-20

**Authors:** Laurie Compère, Marco Sperduti, Thierry Gallarda, Adèle Anssens, Stéphanie Lion, Marion Delhommeau, Pénélope Martinelli, Anne-Dominique Devauchelle, Catherine Oppenheim, Pascale Piolino

**Affiliations:** ^1^Laboratory of Memory and Cognition, Institut de Psychologie, Université Paris Descartes, Sorbonne Paris CitéBoulogne-Billancourt, France; ^2^Center of Psychiatry and Neurosciences, Institut National de la Santé et de la Recherche Médicale UMR S894, Université Paris DescartesParis, France; ^3^Laboratory of Physiopathology of Psychiatric Diseases, Centre Hospitalier Sainte AnneParis, France; ^4^Department of Radiology, Centre de Psychiatrie et Neuroscience, Institut National de la Santé et de la Recherche Médicale U894, Université Paris DescartesParis, France; ^5^Institut Universitaire de FranceParis, France

**Keywords:** autobiographical memory, sex differences, personal semantic memory, emotion, fMRI

## Abstract

Autobiographical memory (AM) underlies the formation and temporal continuity over time of personal identity. The few studies on sex-related differences in AM suggest that men and women adopt different cognitive or emotional strategies when retrieving AMs. However, none of the previous works has taken into account the distinction between episodic autobiographical memory (EAM), consisting in the retrieval of specific events by means of mental time travel, and semantic autobiographical memory (SAM), which stores general personal events. Thus, it remains unclear whether differences in these strategies depend on the nature of the memory content to be retrieved. In the present study we employed functional MRI to examine brain activity underlying potential sex differences in EAM and SAM retrieval focusing on the differences in strategies related to the emotional aspects of memories while controlling for basic cognitive strategies. On the behavioral level, there was no significant sex difference in memory performances or subjective feature ratings of either type of AM. Activations common to men and women during AM retrieval were observed in a typical bilateral network comprising medial and lateral temporal regions, precuneus, occipital cortex as well as prefrontal cortex. Contrast analyses revealed that there was no difference between men and women in the EAM condition. In the SAM condition, women showed an increased activity, compared to men, in the dorsal anterior cingulate cortex, inferior parietal and precentral gyrus. Overall, these findings suggest that differential neural activations reflect sex-specific strategies related to emotional aspects of AMs, particularly regarding SAM. We propose that this pattern of activation during SAM retrieval reflects the cognitive cost linked to emotion regulation strategies recruited by women compared to men. These sex-related differences have interesting implications for understanding psychiatric disorders with differential sex prevalence and in which one of key features is overgenerality in AM.

## Introduction

Several psychiatric diseases such as depression (Weissman, 1977), schizophrenia (Aleman et al., 2003), eating disorders (Fairburn and Harrison, 2003), post-traumatic stress disorder (Breslau, 1997), and autism (Anello et al., 2009) are characterized by different prevalence between women and men. This prevalence could be related to sex differences in memory and other domains of cognition (for a review, see Piefke and Fink, 2005) and especially in autobiographical memory (AM) whose overgenerality is a key feature of depression and post-traumatic stress (Lemogne et al., 2006; Moore and Zoellner, 2007). This difference could also be linked to societal factors such as different education for boys and girls. Indeed, sex-role definitions are culturally mediated (Block, 1973; Reilly et al., 2016) and a growing body of literature provides evidence that social norms and expectations define very precociously how autobiographical memory develops in children and especially the type of information (i.e., women focus more on emotional and interpersonal features while men focus more on agentic features of events) included in the narrative of personal experiences of boys and girls (for a review see Grysman and Hudson, 2013).

Previous studies have shown that men show an advantage on spatial tasks while women show an advantage on verbal ones (Halpern, 2000). Nevertheless, there is growing evidence that sex differences are larger and more complex than was classically thought (for reviews, see Halpern, 2000; Andreano and Cahill, 2009), since differences in performance between the sexes were observed in a number of tasks that are neither purely spatial nor verbal. Data suggest that for some tasks, men and women may use different neural paths to reach the same behavioral outcome (Grabowski et al., 2003; Piefke et al., 2005). In the same vein, there is converging evidence suggesting that sex-related differences may depend on the use of different cognitive strategies (i.e., allocentric or *gestalt* or coordinate strategies for men, and egocentric or serial or categorical ones for women in visuospatial tasks; Sharps et al., 1993; Jordan et al., 2002; Hugdahl et al., 2006). Observed differentiation in cognitive strategies could also be related to sex differences in emotional processing. For instance, men report doing things to distract themselves when they are in a depressed mood, while women report focusing their attention on their mood in similar circumstances (Nolen-Hoeksema, 1987), that could be linked with the greater tendency of women to ruminate (Nolen-Hoeksema et al., 1993). Moreover, neuroimaging studies showed that men presented decreased activity compared to women during the use of emotion regulation strategies in regions such as the amygdala, associated with emotional arousal, and that women recruited to a greater extent prefrontal regions when employing emotion regulation strategies (McRae et al., 2008; Domes et al., 2010). These results suggest that emotion regulation is more effective in men and require more cognitive resources in women. This is congruent with the findings of other studies reporting an overall more direct or faster emotional response in women and a more efficient or automatic emotion regulation in men (for a review see Whittle et al., 2011).

Interestingly, sex differences have been observed in autobiographical memory (AM) which underlies the personal identity and the temporal continuity of an individual by gathering all the information defining oneself over time. AM is a multifaceted construct that involves intermixed spatial and verbal information, controlled research strategies and emotional processes. In this long-term memory system related to the self, three sub-components, corresponding to different levels of abstraction, have been distinguished. The first one, episodic autobiographical memory (EAM, e.g., the performance of Carmen that I went to see at the Opera Garnier in February) concerns unique, specific personal events characterized by both a precise spatial and temporal context and external and internal details remembered vividly, demanding mental time travel and the re-experiencing of the event (Nigro and Neisser, 1983; Rathbone et al., 2009). The highest level of EAM recollection is generally linked to emotional intensity (Talarico et al., 2004), to self-relevance (Martinelli et al., 2013b) and to a first-person perspective (Piolino et al., 2006). The second component, semantic autobiographical memory (SAM), concerns generic (i.e., extended and/or repeated events) personal events (e.g., school trip to Germany; walking my dog every morning) and factual personal knowledge (e.g., where and when I was born; names of personal acquaintances and addresses) that are retrieved without reliving a specific instance (Conway and Pleydell-Pearce, 2000; Piolino et al., 2009; Renoult et al., 2012; Prebble et al., 2013). Generic personal events represent summaries of personal experiences that can be highly self-relevant and are considered to be the most common entry for AM retrieval (Conway and Pleydell-Pearce, 2000). At the most abstract level, self-knowledge concerns personality traits (Klein, 2010), personal attitudes and goals and all other self-concepts that define our identity model (Conway, 2005).

The neural networks underpinning these three levels of self-representations have both common and distinct neural bases (see for a review Martinelli et al., 2013b). In common they are concerned by self-reference that has been associated with greater activity in cortical midline structures, comprising the medial prefrontal cortex (MPFC), the anterior cingulate cortex (ACC), and the posterior cingulate cortex (PCC) (Craik et al., 1999; Fossati et al., 2003; D'Argembeau et al., 2005; Northoff et al., 2006; Gutchess et al., 2007). Additionally, the EAM predominantly activates posterior and limbic regions including the hippocampus, while SAM is associated with anterior activations and also posterior and limbic activations to a lesser degree than EAM (see also Maguire, 2001; Svoboda et al., 2006; Cabeza and St Jacques, 2007; Renoult et al., 2012).

Interestingly, AMs are not simply stored information recorded across the lifespan but rather an organized structure of information about the self that depends on a set of control and executive functions that are responsible for the selection and/or the inhibition of information at both encoding and retrieval (Conway and Pleydell-Pearce, 2000; Piolino et al., 2010). Specifically, episodic details are considered more difficult to recollect compared with semantic elements in the hierarchical constructive model of the self-memory system. Moreover, memories are guided by the current self-concept and the self-coherence principle (e.g., positive self-image; Conway et al., 2004; Conway, 2005). Thus, memories are regularly reconstructed each time they are recalled, in accordance with motivational constraints dictated by the current self-concept (Conway and Dewhurst, 1995), as well as by social expectations (e.g., Sanitioso et al., 1990). Therefore, the constructive nature of AM leaves it malleable and open to motivational factors linked to personal relevant goals, in order to maintain psychological well-being (e.g., positive affect) and avoid unpleasant emotions. Indeed, people report recalling their AMs in everyday life in such a way as to regulate their emotions. Thus, it can be expected that regulatory goals influence the nature and the content of AMs that are more likely to be recalled in a given context (Josephson, 1996; Conway et al., 2004).

Many AM studies in the cognitive domain have focused on sex-related differences. These studies highlight an advantage for women in EAM. Indeed, women demonstrate more specific and detailed evocations than men (Pillemer et al., 2003; Pohl et al., 2005). Women generally access their memories faster (Davis, 1999), date them more accurately (Skowronski et al., 1991), and use more emotional terms in their reports (Friedman and Pines, 1991; Herz and Cupchik, 1992; Fuentes and Desrocher, 2013) during spontaneous retrieval tasks. These findings may suggest a specific advantage for women restricted to retrieving EAM since no advantage is reported for general semantic memory tasks (Herlitz et al., 1997).

To date, two main hypotheses have emerged in attempting to explain sex differences in AM:

- The *cognitive style hypothesis* states that men favor contextual spatial strategies and women verbal strategies when encoding and retrieving personal events (Seidlitz and Diener, 1998; Piefke et al., 2005; St. Jacques et al., 2011). In this approach, the verbal investigation method of autobiographical memory might benefit women;
- The *affect intensity hypothesis* argues that women have superior mnemonic abilities because they experience, encode and remember life events with greater emotional intensity than do men and that women may encode and consolidate life events more deeply than men through more frequent elaborative rehearsals (Fujita et al., 1991).

The results of Fujita et al. (1991)'s study extend to the intersection between the regulation of emotion and memory, suggesting that sex-differences in emotional processing could have significant mnemonic consequences. For instance, research has shown that men tend to express their emotions less than women in accordance with social expectations (Kring and Gordon, 1998). Expressive suppression, corresponding to the inhibition of emotional reactions, is an emotional regulation strategy that can interfere with information encoding or retrieval in memory (Richards and Gross, 2000). Overall, these findings constitute a plausible explanatory hypothesis for the female mnemonic advantage in EAM.

As sex differences are complex, studies in this domain can greatly benefit from neuroimaging. To our knowledge, only three studies have attempted to better understand the neural bases of sex differences in AM, but their results did not clearly disentangle these two hypotheses. All these studies focused on specific memories and highlighted common neural substrates of AM in men and women but also subtle differences. In the first study, participants were asked to report both childhood and recent memories with positive or negative emotional valence from personalized stimuli. The authors reported sex differential brain activations, while the ratings of memories did not show any significant sex-related differences (Piefke et al., 2005). Across all types of AMs investigated, men showed greater activation than women in the left parahippocampal region and women showed larger activation than men in the right dorsolateral prefrontal cortex. In the case of old and negative memories, women showed larger activation than men in the right insular cortex. The differential activations of the parahippocampal and dorsolateral prefrontal cortex were interpreted by the authors as reflecting the use of different strategies to recollect memories of personal events (a strategy relying on spatial context in men and on temporal context in women), thus supporting the cognitive style hypothesis. Given that the insula is not only involved in memory and cognition but also in emotion processing (Phan et al., 2002), this activation in women also provides support for the affect intensity hypothesis. However, what is not discussed by the authors is that the hippocampal region is also recruited to a greater extent in emotional AMs (Addis et al., 2004b) and the dorsolateral prefrontal cortex is also involved in emotion regulation (Goldin et al., 2008). Therefore, these results could be coherent with both the cognitive style and the affect intensity hypotheses.

The results of a more recent study (St. Jacques et al., 2011) have been taken to support the cognitive style hypothesis. While comparable behavioral performances were reported between men and women, men showed greater activations than women in regions linked to the experience of reliving events (hippocampus, retrosplenial, and occipital cortex) during the presentation of visuospatial vs. verbal cues. There was no sex difference with verbal cues. The authors suggested that the manipulation of cues reduced the advantage of women for verbal material. However, from the perspective of the affect intensity hypothesis, it is also interesting to note that decreasing mental imagery (or a third person perspective) is a useful strategy to decrease the emotional intensity of a memory (Holland and Kensinger, 2013), suggesting that women might have used this emotional control strategy to reduce the impact of potentially recalled emotional memories.

Finally, comparing the recollection of various personal events according to their valence, Young et al. (2013) found that women recalled more negative AMs than men but that the groups did not differ in other properties of these memories. Across all valences, the differential activations related to sex involved the dorsolateral prefrontal cortex, dorsal anterior insula and precuneus, which were more activated in women. However, unlike Piefke et al. (2005), the authors interpreted the increased activity in the dorsolateral prefrontal cortex in women, especially when recalling positive AMs, as indicative of a greater engagement of cognitive control to minimize intense emotional responses. Indeed, comparing positive and negative AMs directly, women showed increased activation in the dorsolateral prefrontal cortex for positive memories, while activation in the same region was not modulated by the valence of memories in men. Given that most of the regions showing a greater activation in women are involved in emotional processing and modulation (Phan et al., 2002; Goldin et al., 2008) or in the experience of empathy and compassion (Olson et al., 2007; Lamm and Singer, 2010), the authors suggested that women may have more “social” memories than men. Moreover, the activity in the right hippocampus was increased for both positive and negative memories in men, compared to women. On the contrary, increased hippocampal activity was only observed for positive AMs in women. According to the authors, this reflected an attempt by women at emotion regulation to minimize the emotional intensity of the negative AMs.

Overall, these data support the idea that men and women favor different strategies to retrieve memories of specific personal events. Globally, all the aforementioned results were interpreted as supporting the cognitive style hypothesis, but Young et al. (2013) were the first to suggest that the observed differences may be related to emotional processing or regulation. However, to date, neuroimaging studies on sex-related differences in AM are still scarce and provide inconsistent findings. Therefore, the objective of the present study was to extend the literature on the gender differences in AM neural correlates through two approaches.

First, in order to simplify the inferences on the neural correlates of gender differences in AM recollection we decided to minimize the impact of cognitive strategies in order to focus on the emotion strategies suggested by the affective intensity hypothesis. For this purpose, we constructed a control condition, impersonal scene imagery, that was close to the experimental conditions (i.e., access to information from sentence cues, scene construction, mental imagery), but with one main difference. In the experimental condition, the target of the task was AM (EAM or SAM), whereas in the control condition the target was general semantic memory. We reasoned that a subtraction of the activations from the control condition from those of the experimental conditions would then reveal more specially the brain regions responsible for the emotional aspects of AMs (i.e., emotional processing, involvement of emotion regulation strategies and AM selection/inhibition processes). Since the affect intensity hypothesis suggests that women experience and remember personal events as more emotionally intense than men, we assumed that the activations related to emotional aspects of AMs would be greater in women than in men (e.g., frontal and limbic regions attesting the involvement of emotion regulation strategies and emotion processing respectively).

Second, while all the previous studies investigated sex differences in EAM, we aimed to examine sex differences considering the distinction between specific and generic personal events, i.e., EAM and SAM. Based on previous behavioral findings on the effects of sex on the retrieval of personal specific events, we expected sex differences in the EAM condition (Friedman and Pines, 1991; Herz and Cupchik, 1992; Pillemer et al., 2003; Pohl et al., 2005; Fuentes and Desrocher, 2013). We did not have any strong hypothesis about the SAM condition as generic personal events have never been studied in the context of gender studies; however, based on the absence of sex differences in general semantic memory (Herlitz et al., 1997), we expected no sex difference in the SAM condition.

Thus, contrasting the two AM tasks with the control task, we expected that differences in activation between men and women would inform us on the existence, the magnitude and the nature of strategies linked to emotional aspects of AMs and how regions previously reported in the literature (i.e., the dorsolateral prefrontal cortex, amygdala, hippocampus, precuneus, retrosplenial, and occipital cortices) underlie some sex differences in emotion-related strategies.

## Methods

### Participants

Twenty women and 16 men, all right-handed (according to the Edinburgh Handedness Inventory; Oldfield, 1971) and native French speakers, participated in the study. The two groups were matched for age [women: mean = 47.75 ± 20.53 (min: 25–max: 75), men: mean = 44.25 ± 19.34 (min: 25–max: 72), *t*_(34)_ = 0.52, *p* = 0.60]. All participants gave their informed written consent as required by the local ethics committee (CPP Ile de France 3 n°2687). About one month before the scanning session, participants were tested for exclusion and inclusion criteria: they underwent a medical examination, neuropsychological assessment and completed the Taste and Interest Questionnaire (TIQ) that was employed afterward to create the personal verbal cues used in the scanning session. All were unmedicated and were in good general health as clinically screened by a medical exam. Exclusion criteria included a history of alcohol or substance abuse, head trauma, major disease affecting brain function, neuropsychiatric disorders (tested with the Mini-International Neuropsychiatric Interview, Sheehan et al., 1998), depression (BDI-21, Beck et al., 1988; Bouvard and Cottraux, 1996, cut off score >14: women: 2.85 ± 2.51 and men: 3.00 ± 2.47), abnormal general cognitive functioning as assessed by the Mattis scale (Mattis, 1976, cut-off score lower than 136; women: 141.05 ± 2.95 and men: 141.75 ± 1.88), and abnormal visual mental imagery ability (short form of the Minnesota Paper form Board test: (Vandenberg and Kuse, 1978), lower than 2 points over 5 points); women: 4.30 ±.57 and men: 4.25 ± 1.06).

Moreover, they all performed within their normal age range for verbal memory as assessed by the Grober and Buschke (1987) test [sum of 3 total recalls, delayed total recall; women: 46.35 ± 2.41, 15.85 ±.49 and men: 46.75 ± 3.41, 15.75 ±.77, *t*_(34)_ = −0.41 and 0.47, *p* = 0.68 and *p* = 0.64]. Finally, both groups were matched according to their verbal abilities and crystallized intelligence as assessed by the Mill Hill test (Deltour, 1993; a multiple-choice synonym vocabulary test), [percentile score; women: 53.94 ± 27.29 and men: 60.31 ± 33.75, *t*_(34)_ = −0.62, *p* = 0.53]. The number of years of education was higher for men than for women [women: 13.80 ± 3.05 and men: 16.68 ± 2.98, *t*_(34)_ = −2.85, *p* = 0.007].

### Neuropsychological measures

In order to characterize executive and working memory functions involved in AM search strategies, we administered a brief neuropsychological test battery. We followed the recommendation of Miyake et al. (2000), who proposed the distinction between three elementary executive processes: “shifting,” “updating,” and “inhibition” of inappropriate responses. Consistently, we administered the following standard tests to the participants (Piolino et al., 2010, for details): the trail making test (Reitan, 1958, TMT B-A), running span (Quinette et al., 2006; total score), and the Stroop test (Stroop, 1935; interference score), to assess shifting, updating and inhibition functions, respectively. Other measures of verbal fluency (Cardebat et al., 1990, sum of animal and letter P fluency), and digit span (sum of backward and forward spans, Wechsler, 2000) were administered to assess cognitive control and working memory functions. A multimodal span measured the capacity to integrate what-where-when information (i.e., increasingly long strings of objects associated with a specific spatial context and recall them immediately afterwards in the same order), as this capacity is important during the recollection of episodes.

Otherwise, for the purposes of the study, the two sex groups were also compared on a self-concept scale (adapted from the Tennessee Self-Concept Scale (TSCS); Fitts and Warren, 1996; French version, see Duval et al., 2007; Lalanne et al., 2013; Martinelli et al., 2013a), in order to obtain a measure of positive valence of the self (positive self-image or self-esteem).

### Neuroimaging procedure

#### Visual personalized cues for evoking autobiographical memories

The TIQ (Martinelli et al., 2013a; Sperduti et al., 2013) were submitted to subjects. The aim of this questionnaire was to collect information enabling us to create personalized cues for each participant without directly asking them to describe past memories to avoid the re-encoding of memories (Addis et al., 2004b; Viard et al., 2007). Participants were informed that the purpose of the questionnaire was to obtain a brief description of their personality thanks to information about their principal life interests excluding the recent period of the last 5 years. Participants had no prior knowledge of the aim of the fMRI task, preventing the possibility of participants searching for memories linked to their taste and interests between the two sessions. The TIQ consists of a list of 220 interests including leisure, food, drink, transport, residence, holidays, jobs, and studies. For each item, the participants had to answer whether it was personally relevant or not, rated by 1 and 0 respectively. When an item was relevant, they had to rate how important (from 0 to 10) and frequent (Frequent/Rare) the activity or interest had been in their life. An activity or interest was used as a cue for AM retrieval if it was relevant and important (>5), and frequent (e.g., chess club) for SAM or infrequent (e.g., a trip to Venice) for EAM. Forty-eight personalized cues (2 × 24) were created for each subject with the following structure for EAM—“a unique memory linked to…” and for SAM “a habit of the past linked to…”—followed by the selected items from the TIQ for the respective category.

#### Task design

During scanning, cues were visually presented in white font on a black background projected on a screen viewed by means of a mirror incorporated in the head-coil. E-Prime software (Psychology Software Tools, Inc., Pittsburgh) in combination with the Integrated Functional Imaging System (IFIS) was used for the presentation and timing of stimuli and collection of responses. Responses were made on an MR-compatible two-button box.

**Figure 1:**
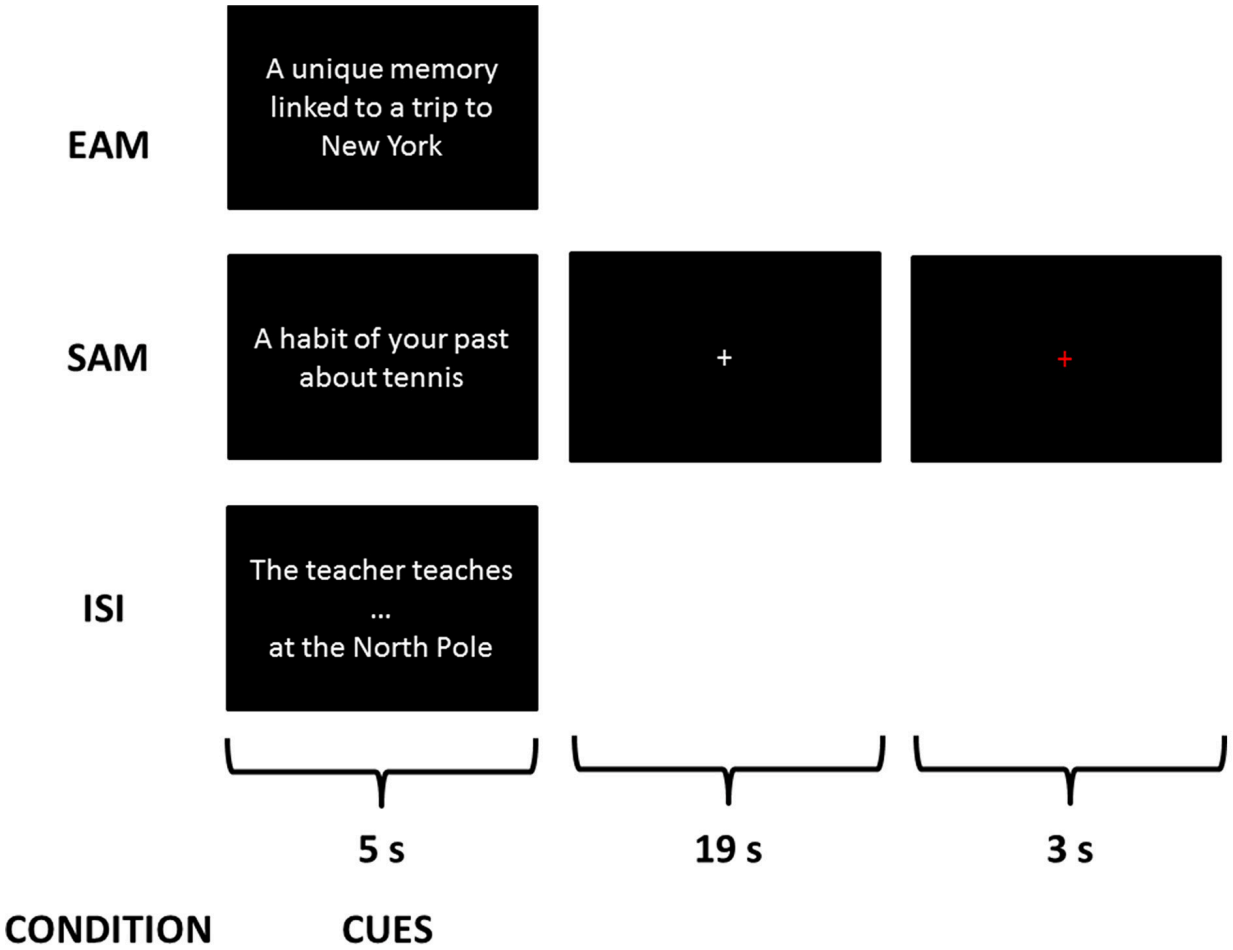
**Examples of a trial of each condition during the scanning session**. This is a fixation cross corresponding to what the participants saw on the screen while performing the exercise.

Three conditions were explained to the subjects consisting in retrieving three different types of information from cues: an EAM condition, a SAM condition and a control condition—impersonal scene imagery (ISI)—(see Figure 1). A training session was proposed with cues different from those used during the scanning session. After instructions, participants were trained on three trials for each condition with the experimenter giving feedback concerning the pertinence of each response.

For EAM and SAM tasks, we used the same instructions as previously published in other AM studies designed to examine the episodic and semantic aspects of AM based on single vs. repeated/extended event retrieval (e.g., Addis et al., 2004a; Levine et al., 2004; Piolino et al., 2010).

For the EAM condition, participants were instructed to mentally recall specific personal events elicited by the cues in the scan. Specific memory was defined as a memory of a single event that occurred at a specific time and place, of short duration, lasting less than 24 h. Participants were instructed to mentally relive personal episodes prompted by cues and to try to retrieve spatiotemporal, affective and perceptual details (such as time, location, perceptions, feelings, scenery, and people present in the scene).

For the SAM condition, they were instructed to recall generic memories of repeated events that occurred several times in the past, a regular activity that used to occur at a routine time and place or a memory of an extended event that may describe a summary of events over several days, weeks, or months without a precise moment in time.

For the control condition, named impersonal scene imagery (ISI), they were instructed to first complete a sentence (e.g., “the teacher teaches…”) with the first word that comes in mind (e.g., “math”) and then to imagine the scene described in the sentence in a peculiar context, the North Pole. This scenario was used due to the non-personal character of the North Pole in order to avoid any indirect reference to autobiographical memory in the control condition. Thus, participants were explicitly instructed to imagine the scenes with no reference to their personal life. The design of such a control condition aimed at controlling processes involved in the two AM retrieval tasks but not related to the self: encoding of new information, access to general semantic knowledge and scene construction.

Participants completed four functional scans, each lasting 9 min 16 s, in a single session. Each functional scan was composed of 18 items (6 EAM, 6 SAM, and 6 ISI) presented in a randomized order within mini-blocks of 3 items of the same condition. Each trial lasted 27 s with the following time-course: the cue was presented for 5 s, followed by a white cross at the center of the screen for 19 s, then the cross turned red for 3 s informing the participants of the end of the present trial and the upcoming of the next one. The duration of 19 s took into account inter-individual variability in accessing AMs, especially regarding EAMs, and allowed each participant to successfully complete the task (Viard et al., 2011). For all conditions, they were instructed to search for information and then to press a button as soon as they gained access to the memory or they completed the sentence and began to visualize the mental images of specific or generic personal scenes or of a non-personal scene in the context of the North Pole.

After the scanning session, participants were asked to recall again each EAM and SAM previously retrieved in the scanner in order to check that memories met minimal criteria for the corresponding category (i.e., EAM: single events, situated in time and place, lasting less than 24 h; e.g., “I remembered the day I spent at Disneyland with my parents shortly before their divorce, it was in summer, 10 years ago”; SAM: repeated or extended events; e.g., “I recalled my riding lessons every Saturday morning,” “I recalled the period of my first job”).

Each EAM or SAM was rated for specificity of episodes and richness of details based on a 7-point scoring grid (Piolino et al., 2009). According to this grid, one point can be given for each of the following categories: the uniqueness (unique “what” vs. multiple), the specificity (shorter than 24 h vs. longer), time situation (“when”), space situation (“where”), contextual non-temporal and non-spatial details (who, how…) and phenomenological details (thoughts, perceptions, emotions). This scale is quite similar to episodic memory scales used for normal subjects and patients in our foregoing studies (Piolino et al., 2002, 2003, 2006; for a review, Piolino et al., 2009) based on previous scales (Baddeley et al., 1986; Kopelman et al., 1989). The first two questions were used to check the minimum criteria for specific or generic AM. The following four questions were then used to measure the level of detail (specific for EAM, generic for SAM). Thus, we recorded (1) the percentage of EAM and SAM (based on minimal criteria), (2) the percentage of EAM meeting stringent criteria (EPI SCORE, i.e., memories with all categories of information), and (3) the number of details for EAM and SAM (max. 4).

In addition to objective measures based on specificity and level of internal and external details, the subjective reports of memories were also assessed using self-evaluations of different phenomenological aspects of memory retrieval which are known to be critical in AM (Piolino et al., 2009). As previously used in our neuroimaging studies of AM (Piolino et al., 2004; Viard et al., 2007), we asked participants to state their self-perspective (Nigro and Neisser, 1983) during each recall under the scan, i.e., no self-perspective, seeing oneself as an observer (third-person perspective), seeing oneself as an actor (first-person perspective), (max. 2 points); valence of memories from very negative (−2) to very positive (2) (max. 2 points); and specifically for EAM, the intensity of the sense of remembering as mental reliving of a specific event on a 6-point scale from very low (0) to very high (5). We added a question on the self-relevance of each memory, asking the participant if the memory was highly relevant to define or explain his/her personality (Singer et al., 2007) (yes/no response, max. 1). For each participant, all the scores were the mean scores across all memories recalled under the scan.

#### Functional imaging

All data were acquired with a 3 T scanner (Discovery MR 750, General Electric Healthcare, Little Chalfont, United Kingdom). The anatomical scan used an inversion recovery 3-D T1-weighted gradient-echo image sequence (*TE* = 4.3 ms, *TR* = 11.2 ms, *TI* = 400 ms, matrix = 384 × 384, slice thickness = 1.2 mm). Functional images were acquired using a gradient echo echoplanar (EPI) sequence (*TE* = 30 ms, *TR* = 2000 ms, flip angle = 90°, matrix = 64 × 64, slice thickness = 3 mm, 42 contiguous sections). The first four volumes of each functional run were discarded in order to allow longitudinal magnetization to approach equilibrium.

#### Statistical analysis of fMRI data

All data were processed using SPM5 software (Statistical Parametric Mapping 5, Welcome Dept. Cognitive Neurology, UK; www.fil.ion.ucl.ac.uk/spm). Standard pre-processing procedures were applied to MRI data. EPI volumes were corrected for slice timing, realigned to the first image, co-registered with the high-resolution T_1_-weighted image and normalized into the Montreal Neurological Institute (MNI) template. Finally, the normalized EPI volumes were smoothed using an isotropic Gaussian kernel filter (5 mm full-width half-maximum).

Only responses meeting the criteria for the three conditions were used for the subsequent analyses. A trial was considered as a hit if (1) the participant had pressed the button during the trial (indicating retrieval) and (2) the description of the memory recalled in the scanner during the debriefing met the criterion for EAM or SAM or the description of ISI in the scanner during the debriefing met the criterion for non-autobiographical reference. Memory retrieval (i.e., access or strategic search phase) was modeled by convolving the time period between cue presentation and subjects' response with the hemodynamic response function (HRF) because this time frame made it possible to model the strategic search and first recall processes. For each subject, the General Linear Model was used to estimate the parameters of interest. Parameters of movement were also included in the model as regressors of no interest. Whole brain *t*-tests were computed to estimate the contrasts of interest for each subject: EAM vs. ISI and SAM vs. ISI. Then, contrasts for each individual were used for second-level analyses.

We first computed a conjunction analysis over the two AM condition contrasts to detect cerebral regions activated in common by the two types of AM retrieval. The voxel-wise statistical threshold was set at *p* < 0.001 (uncorrected), and was corrected for multiple comparison at the cluster level with p(FWE) < 0.05. Valid conjunction inference (Nichols et al., 2005) implies that all the comparisons in the conjunction are individually significant, which corresponds to the valid test for a “logical AND” (i.e., to assess brain areas activated by the EAM task and by the SAM task).

Second, we conducted contrast analyses via a 2 × 2 ANOVA with group (women, men) and condition (EAM, SAM) as between and within factor, respectively. It is interesting to note that we also performed the same analysis with age as a covariate in addition and that this analysis gave the same results. However, since age did not vary significantly across groups, we have not included it in the article (see Supplementary Material). The statistical threshold for these analyses was set at p(FWE) < 0.05 corrected for multiple comparisons at the cluster level, with a voxel-wise threshold at *p* < 0.01 uncorrected. The same ANOVA was computed on an a priori region of interest (ROI) on the left and right hippocampus, a region that is known to be involved in episodic autobiographical retrieval (e.g., Addis et al., 2004a; Viard et al., 2007). This ROI was defined using the template of the Anatomical Automatic Labeling (AAL), (Tzourio-Mazoyer et al., 2002, see **Figure 3**). For this a priori ROI we used a more lenient threshold of *p* < 0.01 uncorrected.

## Results

### Behavioral results

The results of neuropsychological measures and autobiographical scores are reported in Table 1. Since the years of education were higher for men than women, we included this covariate in all the analyses of group effect. They showed no difference between women and men, either for the neuropsychological or for the autobiographical scores. Women and men showed a high percentage of correct trials and a rapid response time that did not differ between the groups.

**Table 1:** **Neuropsychological and autobiographical measures according to the group (mean, ±SD; Min; Max and statistical results)**.

	**Women**	**Men**	**ANCOVA *F*_(1, 33)_**	**Levene Test *F*_(1, 34)_**
**NEUROPSYCHOLOGICAL SCORES**				
INHIB (s)	11.85 (± 7.69;−27.5;1)	10.53 (± 6.94;−29.5;−3.5)	0.86	0.74
TMTB-A (s)	48.15 (± 29.44;−122;−8)	32.87 (± 17.82;−80;−8)	0.94	3.24
R-SPAN	11.55 (± 3.67;5;19)	14.31 (± 3.30;9;20)	0.94	0.16
FLU	56.35 (± 14.24:30:85)	59.94 (± 12.98;43;94)	0.00	1.46
WM	33.89 (± 8.04;22;52)	39.87 (± 8.22;26;53)	0.59	0.02
STBinding	9.00 (± 2.61;5;15)	9.00 (± 3.46;4;18)	1.12	0.93
SCS-Valence	80.42 (± 6.49;71;90)	81.12 (± 4.69;72;90)	0.00	3.28
**AUTOBIOGRAPHICAL SCORES**				
% EAM	91.60 (± 7.61;66.67;100)	88.11 (± 8.39;70.83;100)	0.45	1.18
RT-EAM (s)	2.47 (± 1.45;0.97;7.27)	2.66 (± 1.26;1.18;5.67)	0.48	0.24
%EPI score	77.15 (± 21.07;25;100)	71.96 (± 20.88;20.83;100)	0.07	0.09
EAM-Details (/4)	3.30 (± 0.85;0.60;4)	3.34 (± 0.48;2.10;3.90)	0.02	2.77
Self-perspective (2)	1.21 (± 0.68;0;2)	1.44(± 0.51;0.44;2)	0.31	1.97
Valence	0.69 (± 0.33;−0.14;1.13)	0.75 (± 0.37;0.14;1.47)	0.1	0.57
Self-relevance	0.68 (± 0.21;0.4;1)	0.65(± 0.27;0.15;1)	0.01	2.69
Reliving	2.94 (± 1.44;0;4.91)	2.71 (± 1.68;0;4.11)	1.30	1.42
% SAM	91.67 (± 7.64;70.83;100)	87.59 (± 9.26;66.67;100)	0.61	0.60
RT-SAM (s)	2.50 (± 1.41;0.78;6.01)	2.74 (± 1.34;0.96;5.70)	0.70	0.001
SAM-Details	2.55 (± 1.35;0;4)	2.39 (± 1.17;0;3.95)	0.12	0.36
Self-perspective	1.15 (± 0.72;0;2)	1.06 (± 0.77;0;2)	0.67	0.22
Valence	0.75 (± 0.29;0.16;0.27)	0.71 (± 0.31;0.3;1.29)	0.05	0.09
Self-relevance	0.55 (± 0.29;0;1)	0.41 (± 0.19;0.05;0.71)	0.85	3.28

*NHIB, interference score Stroop, inhibition; TMTB-A, trail making test B-A score, shifting; R-SPAN, running span, updating; FLU, verbal fluency; WM, digit span, working memory; STBinding, multimodal what-where-when span, short-term binding; SCS, self-concept scale; EAM and SAM, percentage of correct responses; RT, response time; EPI, Episodic score of EAM. Results of ANCOVAs (years of education as covariable) and Levene's tests did not show any significant results at statistical threshold of p < 0.05*.

### fMRI results

Given the differential number of years of education between women and men, all the subsequent statistical analyses were corrected for this variable. The resulting activation maps were superimposed onto the MNI template brain of SPM5. Optimal anatomical localization of the significant activations was based on the MNI template brain of SPM5.

#### Conjunction analysis

Relative to the control ISI task, activations common to the two types of AM involved a network, mainly left-lateralized, including the left and right medial prefrontal gyrus, the left posterior cingulate and cuneus/precuneus gyri, the left supramarginal and angular gyri and the left cerebellum (Table 2).

**Table 2:** **Relative increases in brain activity common to all experimental memory conditions across men and women**.

**Region**	***BA***	***k***	***T***	**MNI coordinates**		
				***x***	***y***	***z***
Left precuneus	31	1088	7.50	−6	−54	30
Left cingulate gyrus	31		6.70	−9	−42	35
Left posterior cingulate gyrus	23		6.37	−3	−30	33
Left medial superior frontal gyrus	10	404	5.50	−3	57	9
Left anterior cingulate gyrus	32		5.42	−5	45	18
Right anterior cingulate gyrus	24		4.85	9	35	18
Left angular gyrus	39	111	5.30	−45	−69	33
Left middle temporal gyrus	21		3.73	−54	−57	21
Left cerebellum	17	112	4.46	−6	−75	−15
			4.05	−3	−31	0
Left superior occipital gyrus	17		3.96	−9	−93	3

*Joint effect of all memory conditions vs. control condition (Episodic Condition + Semantic Condition > Control Condition). All reported activations are significant at a voxel-wise threshold of p < 0.001 (uncorrected), corrected for multiple comparisons at the cluster level, p(FWE) < 0.05*.

#### Contrast analysis

The whole brain contrast analysis of each condition and each group indicated several significant activations. For the EAM condition in women, activations were revealed in a large cluster encompassing the right and left medial prefrontal cortex, and posterior regions including the posterior cingulate cortex (PCC), and several regions in the parietal lobe (inferior parietal regions including supramarginal and angular gyri). For the men, we reported activations restricted to a left-lateralized network encompassing the PCC, the precuneus and the inferior frontal gyrus. For the SAM condition in women, the right middle frontal gyrus, bilateral PCC, left (inferior, angular, and supramarginal) parietal regions, bilateral occipital regions (lingual gyrus) and the right and left thalamus were significantly activated. For the men, we reported a similar pattern of activations encompassing the right middle frontal and left posterior regions but less extended (e.g., no occipital activations), and additional activations in the superior and middle temporal gyrus were reported. The list of local activation maxima for each condition in both groups using cluster level correction for multiple comparisons is reported in Table 3.

**Table 3:** **Contrast of each memory condition < control condition for each group**.

**Region**	***BA***	***k***	***T***	**MNI coordinates**		
				***x***	***y***	***z***
**MEN EAM**						
Left calcarine gyrus	30	10999	8.24	−12	−54	12
Left precuneus	7		7.94	−6	−54	30
Left cuneus	23		7.84	−9	−60	21
Left insula	48	50	5.58	−30	15	−12
**MEN SAM**						
Left calcarine gyrus	30	6649	8.71	−12	−54	12
Left precuneus	30		7.85	−12	−50	21
Left angular gyrus	39		7.53	−48	−55	33
Left superior temporal gyrus	22	86	5.88	−54	−6	−9
Left middle temporal gyrus	20		4.43	−51	−18	−12
Left middle temporal gyrus	20		4.21	−50	−21	−9
Left precuneus	30	61	5.07	0	−54	−30
Right middle frontal gyrus	44	161	4.36	45	27	36
Right middle frontal gyrus	9		4.24	33	18	48
Right middle frontal gyrus	46		4.16	42	39	30
**WOMEN EAM**						
Left precuneus	23	4820	9.61	−6	−57	24
Right calcarine gyrus	30		7.88	5	−54	15
Right superior medial frontal gyrus	10		7.74	3	57	6
Left angular gyrus	39	221	6.12	−45	−69	30
Left angular gyrus	7		4.42	−39	−69	45
Left angular gyrus	39		4.24	−39	−51	27
Right angular gyrus	7	149	4.88	39	−63	36
Right angular gyrus	39		4.34	48	−55	36
Right middle temporal gyrus	21		4.21	54	−57	21
Right middle frontal gyrus	48	50	4.82	36	30	24
Left middle frontal gyrus	9	51	4.56	−18	30	36
**WOMEN SAM**						
Left posterior cingulate gyrus	23	3848	7.97	−9	−48	27
Left cingulate gyrus	23		7.80	−9	−39	35
Right posterior cingulate gyrus	23		7.68	9	−42	30
Left angular gyrus	39	148	5.31	−45	−69	30
Left angular gyrus	39		4.84	−42	−69	42
Left middle temporal gyrus	21		3.87	−54	−57	21
Right frontal superior gyrus	6	84	5.24	24	−9	66
Right middle frontal gyrus	6		3.91	39	−5	60
Right superior motor area	6		3.45	12	−12	63
Left extra-nuclear		68	5.19	−21	−12	12
Left thalamus	Lateral dorsal nucleus		3.68	−12	−18	18
Left calcarine gyrus	18	250	5.09	−12	−93	0
Right lingual gyrus	17		5.06	6	−81	−9
Left cerbellum	18		4.76	−6	−78	−15
Right middle frontal gyrus	48	291	5.05	36	33	24
Right middle frontal gyrus	48		5.03	30	21	35
Right frontal inferior opercularis	44		4.65	36	12	33
Right caudate nuclear	48	156	4.85	24	−12	21
Right thalamus	Ventral anterior nucleus		4.58	6	−9	6
Right caudate nuclear			4.32	12	0	6

*Activations are significant at a voxel-wise threshold of p < 0.01 (uncorrected), corrected for multiple comparisons at the cluster level, p(FWE) < 0.05*.

The results of the mixed 2 × 2 ANCOVA with group (women/men) as between subject factor and condition (EAM and SAM) as within subject factor revealed neither a main effect of group, nor a main effect of condition. Nevertheless, we found a significant interaction between the two factors in the dorsal anterior cingulate cortex (dACC, BA24), the left precentral cortex (BA 6), and the left inferior parietal gyrus (IPG, BA 3), see Table 4.

**Table 4:** **Regions showing an interaction between group and condition**.

**Region**	***BA***	***k***	***F***	**MNI coordinates**		
				***x***	***y***	***z***
Left precentral gyrus	6	610	25.45	−30	−3	36
Left inferior parietal gyrus	3		19.17	−42	−27	45
Right dorsal anterior cingulate cortex	24		13.56	9	18	30

*All reported activations are significant at a voxel-wise threshold of p < 0.01 (uncorrected), corrected for multiple comparisons at the cluster level, p(FWE) < 0.05*.

*Post-hoc* comparisons on signal change showed that activity in the dACC was greater in the EAM compared to the SAM condition in men (*p* = 0.01) and that there was no difference between the two conditions in women (*p* = 0.22). Activity in the dACC was greater in women compared to men (*p* = 0.03) in the SAM condition, and there was no group difference in the EAM condition (*p* = 0.95).

**Figure 2:**
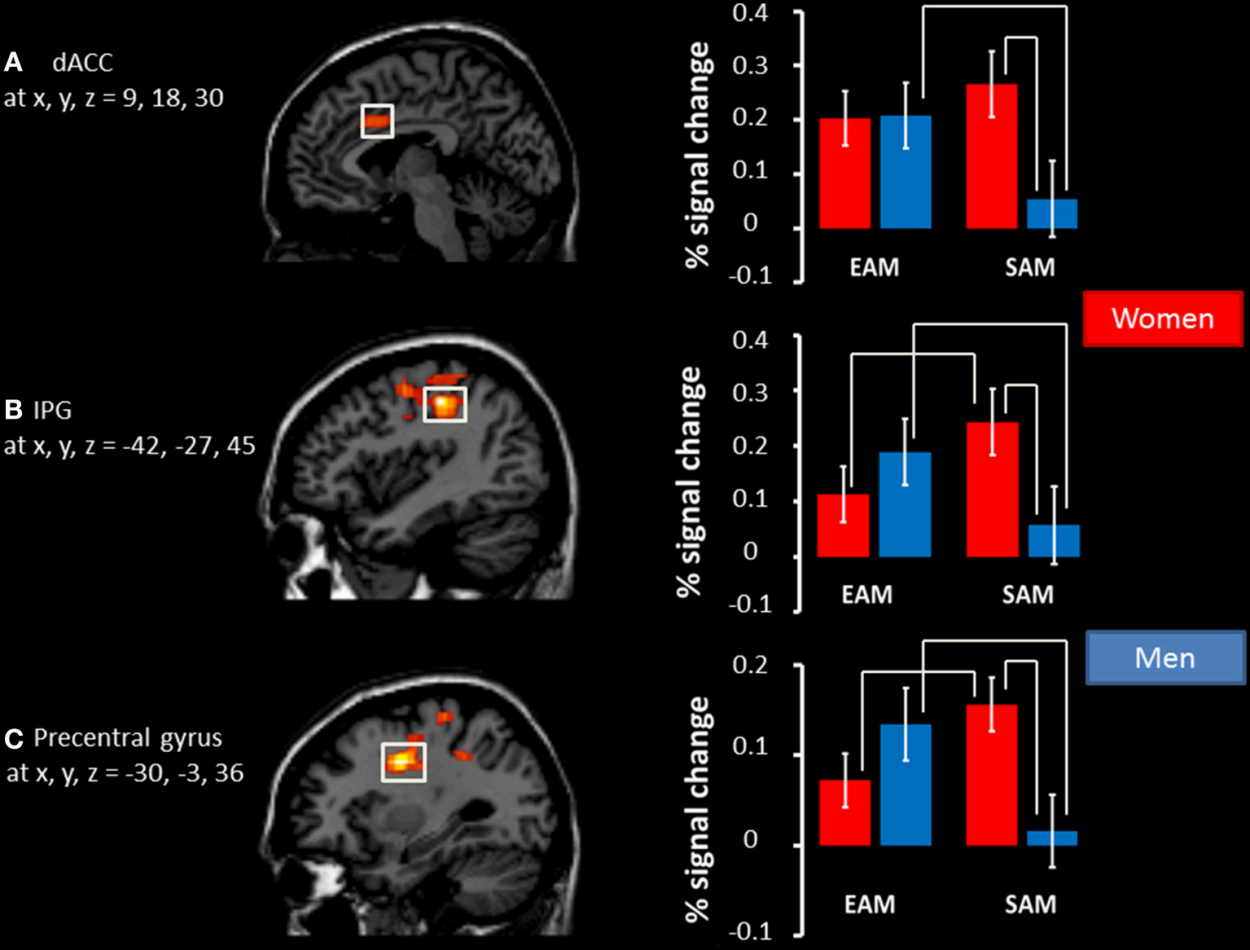
**Significant interactions between condition (EAM/SAM) and group (men/women)**. Left panel: regions showing a significant interaction depicted as voxel *F*-values corresponding to *p* < 0.01 (uncorrected), corrected for multiple comparisons at the cluster level, p(FWE) < 0.05, superimposed on the template. Right panel: The percentage signal change associated with each adjacent image in EAM and SAM in **(A)** the right dACC, **(B)** the left IPG, and **(C)** the left precentral gyrus. Coordinates interpreted as indicated in the legend for Table 4. The significant results of post hoc comparisons (*p* < 0.05) are indicated by the white bars above the histograms.

*Post-hoc* comparisons on signal change showed that activity in the precentral gyrus and IPG was greater in the EAM compared to the SAM condition in men (*p* = 0.03 and *p* = 0.002) and that the opposite pattern was evident in women (*p* = 0.02 and *p* = 0.01). Moreover, activity in both regions was greater in women compared to men (*p* = 0.03 and *p* = 0.01) in the SAM condition, and there was no group difference in the EAM condition (*p* = 0.39 and *p* = 0.25), see Figure 2.

**Figure 3:**
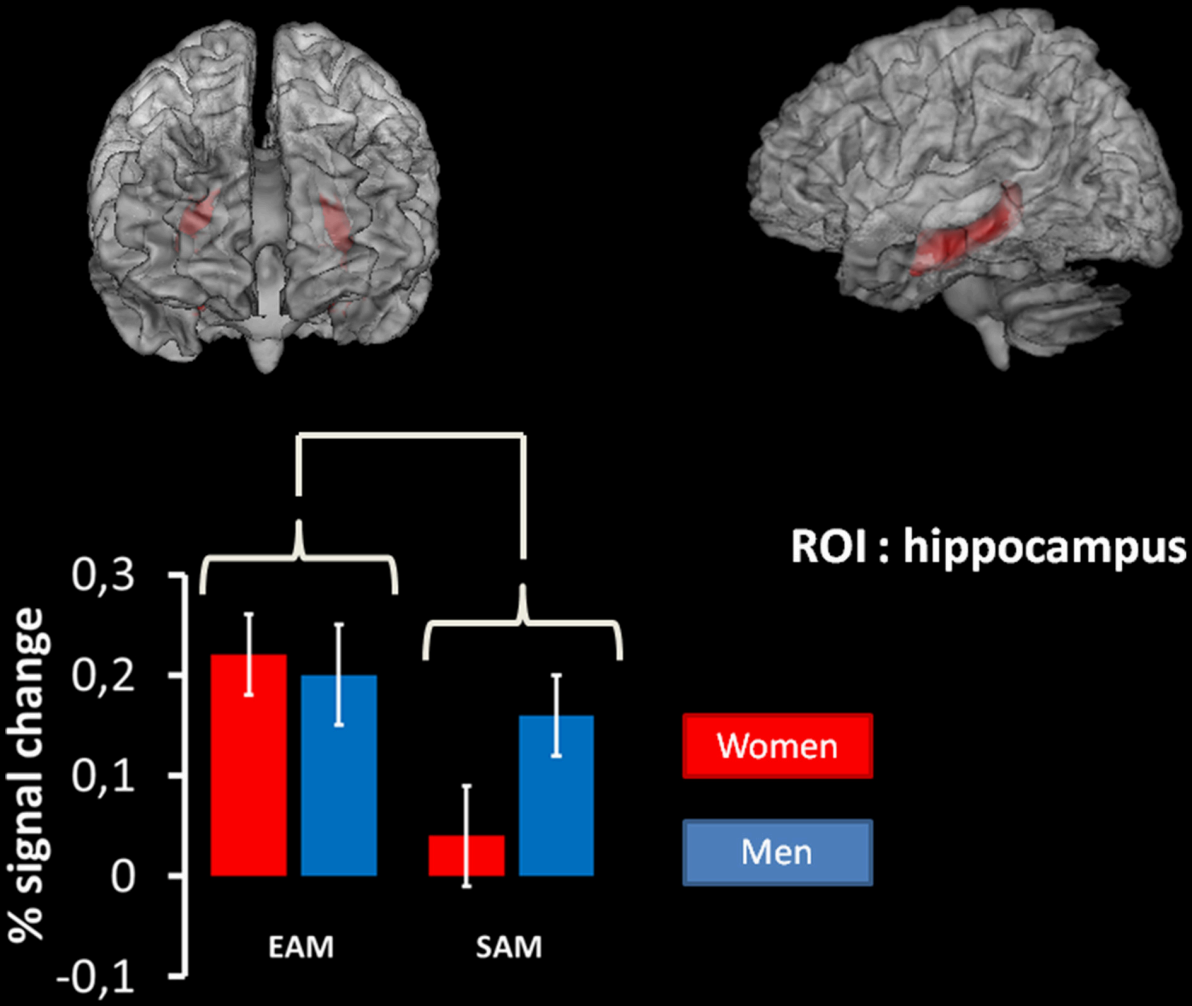
**Results of the 2 × 2 ANOVA with condition (EAM/SAM) and group (men/women) as factors in the right and left hippocampus using region-of-interest analyses**. At the top: region of interest used. ROI are superimposed on an MNI T1 template. The results of the mixed 2 × 2 ANCOVA with group (women/men) as between subject factor and condition (EAM and SAM) as within subject factor on the ROI in the hippocampus revealed a significant main effect of the condition (*p* < 0.01, coordinates of local maxima: *x* = 33; *y* = –21; *z* = –9). At the bottom: plots represent percentage of signal change in right and left hippocampus for each condition of interest in each group.

The same analysis on the ROI in the hippocampus, revealed a significant main effect of the condition (*p* < 0.01, coordinates of local maxima: *x* = 33; *y* = –21; *z* = –9). This effect was due to the fact that this structure was more activated in the EAM compared to the SAM condition irrespective of the group (Figure 3).

## Discussion

The aim of this study was to investigate the sex differences in autobiographical memory distinguishing between EAM and SAM and to concentrate on the affect intensity hypothesis. Our data show evidence for both common and differential neural mechanisms underlying episodic and semantic autobiographical retrieval in men and women.

Across sexes and memory conditions (EAM, SAM), activations were observed in regions that had previously been identified as core components of the AM network (Maguire, 2001; Svoboda et al., 2006; Cabeza and St Jacques, 2007; Martinelli et al., 2013b), including the medial prefrontal cortex, precuneus, cingulate, temporo-parietal, occipital cortices and cerebellum. This finding confirms that compared to the control ISI condition, the two AM conditions involved self-referencing linked to cerebral cortical midline structures, including the medial prefrontal cortex, anterior and posterior cingulate and medial parietal cortex (Northoff et al., 2006; Buckner and Carroll, 2007). Moreover, a ROI analysis on the hippocampus showed that this structure was more active during the recovery of specific autobiographical events compared to general ones regardless of the group. This is in keeping with previous studies showing a disengagement of the hippocampus with the semantization of EAMs and the retrieval of SAMs (Moscovitch et al., 2005; Martinelli et al., 2013b). Interestingly, we showed that this disengagement applied to both men and women.

Behavioral comparisons showed no differences between men and women either in terms of feature memories recollected in each condition or of performances in the different neuropsychological tests (see Table 1). This contrasts with previous behavioral studies revealing sex differences in EAM but it could reflect the fact that sex differences are less evident when using questionnaires and rating scales as dependent variables instead of content analysis of autobiographical narratives (Grysman and Hudson, 2013). Moreover, Grysman and Hudson (2013) suggest that behavioral differences highlighted in autobiographical memory literature are due to a communication mode whereby men and women prefer to include different types of information in their story. However, when contextual demands do not favor this tendency, men are able to produce autobiographical narratives as elaborate and self-reflexive as women when they interact with an intimate partner or when writing alone (Aukett et al., 1988; Grysman and Hudson, 2013). When recalling memories in the scanner, participants were alone and therefore certainly not in a social context in which this sort of information would be exercised and therefore recalled all episodic details available. This could probably explain the absence of difference in EAM retrieval between women and men in the present study. Nevertheless, sex differences were evident in the pattern of brain activation when comparing EAM and SAM, since comparable behavioral performances were accompanied by differential brain activation. This finding is consistent with prior neuroimaging studies (Piefke et al., 2005; St. Jacques et al., 2011), but it extends them to SAMs.

Although there were no sex differences in the ratings of EAM and SAM features and response times under the scan, our neuroimaging protocol was designed to unravel differential retrieval strategies. As we subtracted most basic cognitive strategies thanks to a special impersonal scene imagery task requiring access to semantic knowledge through the completion of a sentence and scene construction which allowed basic verbal access and visuospatial construction strategies to be controlled, we were able to investigate more specifically emotional aspects of the AM retrieval (i.e., emotional processing, involvement of emotion regulation strategies) in order to focus on the affect intensity hypothesis. The sex differences in brain activations were detected in the dACC, the left IPG and the left precentral gyrus. This differed from previous studies showing main differences in the dorsolateral prefrontal cortex, amygdala, hippocampus, precuneus, retrosplenial and occipital cortices (Piefke et al., 2005; St. Jacques et al., 2011; Young et al., 2013). This is likely due to differences in the method used in comparison to previous studies: investigation of EAM and SAM and a different control task. Although these results need to be replicated, we suggest that the differential pattern of activation between men and women, when comparing the two memory conditions (EAM and SAM), could be coherent with the affect intensity hypothesis, whereas the pattern of brain activity reported in previous studies is more likely interpretable in the framework of the cognitive style hypothesis. It is also important to note that the present findings remarkably concerned only memories that met strict criteria of EAMs and SAMs (response under the scanner and post-scan evaluation), so in other words excluded overgeneral recalls in the EAM task or specific recalls in the SAM task. Therefore, it worth mentioning that given these criteria, the present study concerns the analysis of emotional processes associated with successful recovery strategies.

The dACC is part of the medial cortical midline structures (CMS) (Northoff and Bermpohl, 2004; Northoff et al., 2011) associated with self-referencing, but it has been associated with a variety of affective, cognitive, sensory, motor and especially executive functions (Maddock, 1999) and is part of the commonly activated regions during working memory, episodic memory and semantic memory tasks (Nyberg et al., 2003). More precisely, the ACC can be further divided into two major sections responsible for cognitive and emotional processing: the ventral subdivision of the ACC is more involved in affective processing whereas the dorsal subdivision is more involved in cognitive processing (Stevens, 2014). While an involvement of the ventral subdivision of the ACC could be expected, in keeping with the affect intensity hypothesis, the findings revealed activations in the dorsal subdivision of the ACC. We suggest that this is nevertheless compatible with the affect intensity hypothesis.

Indeed, even though the activation pattern in men showing increased activation during EAM vs. SAM in dACC is consistent with its role in cognitive control and effortful task completion (Carter et al., 1998; Dehaene et al., 1998; MacDonald et al., 2000), the activation pattern in women in the dACC suggested that they exercised similar cognitive control during EAM and SAM tasks. This result could suggest that the task of SAM was more difficult to achieve for women than men. However, since both groups have similar behavioral performances, it is more likely that these results reflect the establishment of emotion regulation strategies. Indeed, several emotion regulation strategies are associated with activations of the dorsal part of the ACC (Stevens, 2014). Moreover, in Young et al. (2013), women showed greater BOLD activity than men in the dorsolateral prefrontal cortex which is known to be connected with the dACC (Stevens, 2014), and this result was interpreted by the authors as indicating greater cognitive control to minimize intense emotional responses. Therefore, a more likely explanation is that although the activity in this brain region might contribute to specific needs of cognitive control in EAM (compared to the control task), its activity may specifically underlie the establishment of emotion regulation strategies, particularly in women. Accordingly, the present results regarding SAM are more supportive of the affect intensity hypothesis, but further research is needed to determine the relationship between SAM and emotion-related processes.

In women, this activation is present regardless of the condition and suggests the establishment of emotion regulation strategies whatever the level of abstraction of autobiographical event memory, while, in men, this activation is reduced in SAM. If we accept the interpretation that the engagement of this area probably reflects the use of emotion regulation strategies, this suggests that women engage emotion regulation strategies regardless of the degree of abstraction of autobiographical memory, while men are less prone to make use of these mechanisms in SAM.

SAMs are investigated in this study as memories for generic events, namely, an amalgam of common details of repeated occurrences of similar or time-extended events. Therefore, memory for unique (EAM) and generic (SAM) events relies on different weightings of the same component processes: they both include visual imagery and contextual information (Renoult et al., 2012) except that the generic events are characterized by a decrease in uniqueness and specific details (Holland and Kensinger, 2013). The transformation of unique events into generic events in memory is achieved via semanticization processes (i.e., the extraction of similarities across many events) that summarize the initially episodic details including perceptual and affective features. This process has been shown to be a useful strategy to decrease the emotional intensity of a memory (Holland and Kensinger, 2013). Thus, our findings could suggest that the semanticization process is more effective in men that in women, and that it may be especially beneficial to men since they integrate fewer emotional characteristics than women do. Accordingly, Grysman and Hudson (2013) showed that women tend to include more elaborations about thoughts and feelings at the time of the event, while men detail more agentic components and therefore factual consequences. Following the aforementioned findings, we thus propose that generic memories are accompanied by a more direct emotional response in women compared to men and thus require more emotion regulation strategies because during the semantization process the sorting of information stored in memory about the repeated or extended events differ in men and women. It is also important to note that we selected personalized cues for generic events that were as self-relevant and important as personalized cues for specific events, which may have favored the recall of particularly emotional and self-relevant generic events and this, especially since the repetition of these events make them particularly self-relevant. This hypothesis, however, needs to be confirmed by future behavioral and neuroimaging studies investigating the impact of narrative modes in sex differences, especially by taking into account the distinction between EAM and SAM.

Regarding IPG, the neuroimaging literature indicates that the parietal lobe is important in episodic memory retrieval (Berryhill et al., 2007) in tasks using both laboratory-based (e.g., Wiggs et al., 1999; Sestieri et al., 2011) and autobiographical material (e.g., Fink et al., 1996; Martinelli et al., 2013b). The IPG shows, in particular, greater activity for autonoetic consciousness (i.e., retrieval accompanied by sense of re-experiencing vivid episodic details) than for noetic consciousness (i.e., the sense of knowing that an item occurred in the past in the absence of specific details from the encoding context; Henson et al., 1999; Eldridge et al., 2000; Wheeler and Buckner, 2004; Yonelinas et al., 2005; Daselaar et al., 2006). The link between the IPG and memory revival fits well with the data for men, whose activation was greater in EAM than SAM. However, similar to dACC, activity was greater in women compared to men in the SAM condition and there was no group difference in the EAM condition. Therefore, the pattern of activation observed in SAM may rather be linked with the results reported by Sajonz et al. (2010) showing that the IPG is particularly involved in self-referential processes, or with studies on emotional regulation showing its involvement in the establishment of emotion regulation strategies (e.g., McRae et al., 2008). This could be coherent with the proposal that women are more likely to elaborate about thoughts and feeling than men using spontaneous recall tasks (Grysman and Hudson, 2013). Thus, these results suggest that activation in this region may be indicative of a cognitive cost of contextual information recollection in men or may rather be associated with introspection or emotion regulation strategies especially in women and these findings might be in line with our precedent proposal that SAM retrieval is associated with more emotion regulation strategies in women compared to men.

The same pattern of activation was observed in the precentral cortex. Despite activation in AM (Maddock et al., 2001; Gardini et al., 2006), its precise role in memory retrieval is rarely discussed. Due to its implication in motor processes, it could subserve motor actions imagery, suggesting that SAM concerned more generic actions in women than in men (qualitatively, generalized memories often concerned trips or repetitive actions). Moreover, this hypothesis is further supported by the fact that some studies evidenced that AM retrieval may concern the involvement of the body in both sensing and acting (Dijkstra et al., 2007). These findings open new avenues of research exploring the neural basis of agency in AM in men and women.

One limitation of our study is the small sample size and the variability of the participants' age in each group. Therefore, it would be interesting in future studies to have a sample size large enough to assess both the effect of age and the sex-related differences in autobiographical memory. Indeed, the question of the evolution of sex differences during aging is a point that is currently seldom addressed in the AM literature (Piefke and Fink, 2005; Grysman and Hudson, 2013) and yet interesting since it is known that EAM declines in favor of SAM retrieval in aging (Levine et al., 2002; Piolino et al., 2002, 2006, 2010). Moreover, the present analyses focused on sex-related differences between individuals in memory retrieval of personal events (either specific or generic), but did not examine those differences in other aspects of SAM (e.g., factual personal knowledge) or more conceptual aspects of self (Conway, 2005; Klein et al., 2013; Martinelli et al., 2013b). All these semantic aspects are more condensed and stable representation of the self-referential information than EAM that grounds the coherence of personal identity over time (Conway et al., 2004). We proposed that SAM retrieval is associated with more emotional features and hence that this might involve more effortful emotion regulation strategies in women compared to men, thus it would be interesting to test if this can be extended to all kinds of personal semantic memory or this is specific to generic personal memories. We are aware that AM is a multifaceted area of human cognition at the crossroads of genetic, neurobiological, social, emotional and personal (such as self-consciousness, self-concept, goals and personal meanings) influences (Conway et al., 1993; Davis, 1999; D'Argembeau et al., 2014) which should be investigated in future studies. In addition, further research is needed using a psychobiological approach to sex differences (Tabatadze et al., 2015) in the multifaceted self, assessing different self-contents and self-related processes (Damasio, 1999; Gallagher, 2000; Legrand and Ruby, 2009; Klein, 2010; Northoff et al., 2011). Also, it would be interesting for further research to better control between-subjects differences by performing a qualitative analysis of the content of memories and their valence, as has been done in other AM studies (e.g., Blagov and Singer, 2004; Young et al., 2013). In addition, some of our interpretations about sex-related differences in strategies remain speculative and need to be confirmed by questionnaires to identify what types of emotional regulation are favored by participants. Moreover, as some data suggest a link between emotional strategies and social expectations (Kring and Gordon, 1998), it would be interesting to look beyond sex in future studies and to attempt to define gender identity as the feeling of belonging to a gender to better determine the origin of these differences in terms of social, individual or biological aspects. Indeed, whatever they are, these differences need to be better identified and explained as they could certainly put us on the track of the reasons behind the masculine or feminine prevalence of certain diseases, some examples being depressive and post-traumatic stress disorders whose prevalence is greater in women and in which key features are overgenerality and intrusive memories in AM (Lemogne et al., 2006; Moore and Zoellner, 2007).

Overall, the present study, investigating for the first time sex differences in the distinction between episodic and semantic AM (based on the distinction between specific and generic event memories), suggests that the cognitive style hypothesis alone does not account for all the differences related to sex in AM and that the use of different strategies in AM retrieval could also be related to emotional aspects of memories. Although further studies are necessary, interestingly, this study highlight sex-difference in SAM related to emotional processes.

## Author contributions

LC, PP, MS wrote the article. LC, MS, AD, PM, SL, CO did the neuroimaging exams, data processing and data analyses. PP, LC, TG conceptualized the experiment. AA, MD took care of clinical assessment. All the authors contributed to the final draft of the article.

### Conflict of interest statement

The authors declare that the research was conducted in the absence of any commercial or financial relationships that could be construed as a potential conflict of interest.
