# Supplementary material for: Sex Differences in the Neural Correlates of Specific and General Autobiographical Memory
Source: Front Hum Neurosci. 2016 Jun 20;10:285. doi: 10.3389/fnhum.2016.00285 (PMC4913091; doi:10.3389/fnhum.2016.00285)
Supplement: Supplementary file 1 [file Table1.DOCX]

***Supplementary Material:***

**Sex differences in the neural correlates of specific and general autobiographical memory**

[Laurie Compère](http://frontiersin.org/Community/WhosWhoActivity.aspx?sname=LaurieComp%C3%A8re&UID=95002), [Marco Sperduti](http://frontiersin.org/Community/WhosWhoActivity.aspx?sname=MarcoSperduti&UID=40581), [Thierry Gallarda](http://frontiersin.org/Community/WhosWhoActivity.aspx?sname=THIERRYGALLARDA&UID=274122), Adèle Anssens, Stéphanie Lion, [Marion Delhommeau](http://frontiersin.org/Community/WhosWhoActivity.aspx?sname=MarionDelhommeau&UID=184923), Pénélope Martinelli, Anne-Dominique Devauchelle, Catherine Oppenheim and [Pascale Piolino](http://frontiersin.org/Community/WhosWhoActivity.aspx?sname=PascalePiolino&UID=63425)

1. **SUPPLEMENTARY DATA**

**Table: Regions where hemodynamic activity differed significantly in the interaction Episodic vs. Semantic AMs * Male vs. Female with age as covariate**

|  |  |  |  |  | **MNI coordinates** | | |
| --- | --- | --- | --- | --- | --- | --- | --- |
|  | **Region** | **BA** | **k** | **F** | **x** | **y** | **z** |
|  | Left Precentral Gyrus | 6 | 610 | 25.45 | -30 | -3 | 36 |
|  | Left Inferior Parietal Gyrus | 3 |  | 20.22 | -39 | -27 | 45 |
|  | Right Dorsal Anterior Cingulate Cortex | 24 |  | 13.30 | 9 | 18 | 30 |
|  | | | | | | | |

Interaction effect of Episodic vs. Semantic AMs * Male vs. Female. All reported activations are significant at a voxel-wise threshold of p<0.01 (uncorrected), corrected for multiple comparisons at the cluster level, p(FWE)<0.05.
